# *ETV6-ACSL6* fusion gene in myeloid neoplasms: clinical spectrum, current practice, and outcomes

**DOI:** 10.1186/s13023-020-01478-6

**Published:** 2020-07-28

**Authors:** Xia Wu, Hao Cai, Yu Qiu, Jian Li, Dao-bin Zhou, Xin-xin Cao

**Affiliations:** Department of Hematology, Peking Union Medical College Hospital, Peking Union Medical College and Chinese Academy of Medical Sciences, Beijing, China

**Keywords:** *ETV6-ACSL6* fusion gene, Chronic eosinophilic leukemia, Myelodysplastic syndrome, Myeloproliferative neoplasms

## Abstract

**Background:**

*ETV6-ACSL6* is a fusion gene rarely reported in myeloid malignancies, and its clinical characteristics, proper treatment strategies, and effect on prognosis are poorly understood.

**Results:**

Sixteen patients with the *ETV6-ACSL6* fusion gene were identified, with a median age of 50 years. Twelve patients were male. Clinical diagnoses included chronic eosinophilic leukemia, not otherwise specified, acute myeloid leukemia, and other types of myeloproliferative and myelodysplastic disorders. Ten out of 12 patients had increased levels of eosinophils, and four out of five had increased levels of basophils in peripheral blood. Treatment with tyrosine kinase inhibitors was ineffective. The prognosis of the patients was poor, with seven patients dying within 1 year.

**Conclusions:**

Patients with the *ETV6-ACSL6* fusion gene mainly present with myeloproliferative and myelodysplastic disorders, typically with increased eosinophils and/or basophils and poor survival. Intensive therapies such as allogenic stem cell transplantation should be an initial consideration for eligible patients.

## Background

Translocations involving band 12p13 are frequently seen in myeloid malignancies, through which *ETV6* is frequently rearranged [1, 2]. However, fusion of *ETV6* with *ACSL6* is very rarely reported, and the clinical characteristics, proper treatment strategies, and effect on prognosis are poorly understood [3]. The prognosis of patients with the *ETV6-ACSL6* fusion gene is thought to be rather poor, with most patients surviving less than a year [3]. To expand the current knowledge of the *ETV6-ACSL6* fusion gene, we reported a patient with the *ETV6-ACSL6* fusion gene who presented with chronic eosinophilic leukemia, not otherwise specified (NOS), who importantly received proper treatment regimens and consequently had a good prognosis. In addition, we searched the literature for all cases involving the *ETV6-ACSL6* fusion gene, analyzed the data, and depicted the basic clinical characteristics, current treatment options, and outcomes of the disease.

## Methods

### Case presentation

**Fig. 1:**
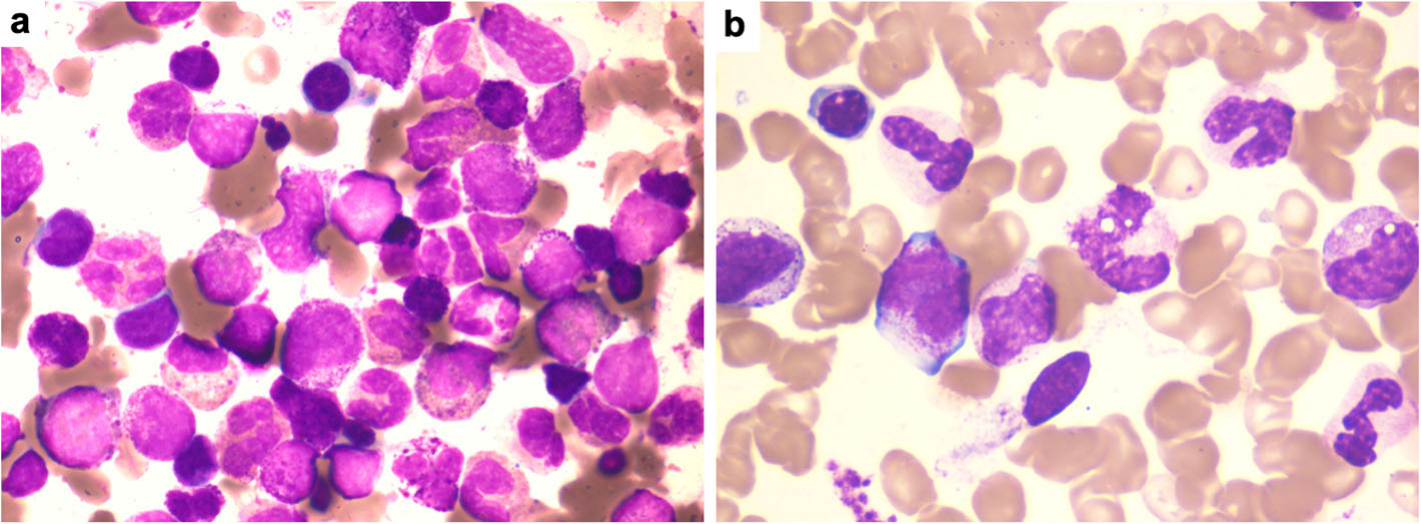
Bone marrow histology in the present case. **a** Bone marrow smear at diagnosis (× 1000). **b** Bone marrow smear after haploidentical stem cell transplantation (× 1000)

A 37-year-old woman with a 16-year history of well-controlled ulcerative colitis presented to Peking Union Medical College Hospital with a 4-week history of continuous fever. Her maximum body temperature was 39 °C. She also had epistaxis and sweating and gradually developed disseminated 2–5 mm papules and nodules over the abdomen, back, and four limbs, with a 7-kg weight loss. She did not report any other symptoms. Family history indicated that three out of four of her mother’s uncles and her grandmother had gastrointestinal tumors. On admission, her body temperature was 38.5 °C; positive physical signs included disseminated reddish to brown itchy papules and nodules over the whole body, sternum tenderness, a liver 3 cm below the costal margin, and a spleen 1 cm below the costal margin. Laboratory findings were as follows. Routine blood tests showed that the white blood cell (WBC) count was 42.4 × 10^9^/L, the platelet count was 24 × 10^9^/L, and hemoglobin was 70 g/L. The peripheral blood smear revealed 53% eosinophils and 28% basophils. The bone marrow smear revealed markedly hypercellular bone marrow with 7% myeloblasts as well as significantly increased eosinophil and eosinophil precursors at different stages (39.5%) and increased basophil and basophil precursors (21.5%) (Fig. 1). With the AML/MDS/MPN Sequencing Panel (Rightongene), we performed targeted amplicon sequencing on the Illumina MiSeq sequencing platform (Illumina, San Diego, CA, USA) using the patient’s peripheral blood and found mutations in *DNMT3A*, *NPM1*, and *GATA2*; meanwhile, no abnormalities in the rest 31 genes of the panel, including *PDGFRA*, *PDGFRB*, *FGFR1*, and *JAK2* were noticed [4]. Paired-end, 101-bp RNA sequencing of over 900 fusion genes using the Illumina NextSeq 500 instrument (Illumina, San Diego, CA, USA) was performed commercially by USCI Medical Laboratory Company Limited. An out-of-frame fusion of exon 1 of *ETV6* to exon 2 of *ACSL6* with a fusion transcript abundance of 17.83% was identified; meanwhile, the rearrangement of other fusion genes, including *FIP1L1-PDGFRA*, *IGH-FGFR3*, *TEL-FGFR3*, and *BCR-ABL*, was not detected. Additionally, oral mucosal epithelial cells were obtained from the patient and her mother for germline testing, and the *GATA2* mutation was not detected by Sanger sequencing using an ABI 3500 DX Genetic Analyzer (Applied Biosystems Inc., Foster City, CA, USA). After admission, the patient’s fever persisted with a maximum body temperature of 39.0 °C, while her peripheral WBC count rose to 82.1 × 10^9^/L. She was diagnosed with chronic eosinophilic leukemia, NOS, and standard induction chemotherapy with 100 mg/m^2^ cytarabine (subcutaneously, every 12 h, days 1–7) and 60 mg/m^2^/d daunorubicin (intravenously, days 1–3) was used. Adverse events were not observed except for bone marrow hypocellularity and febrile neutropenia. Two weeks after the start of chemotherapy, her skin lesions diminished, and she was no longer feverish; her liver and spleen were untouchable below the costal margin. The level of eosinophils was decreased on a repeat bone marrow smear and was only slightly increased overall (5.5%), with 0.5% myeloblasts. A test for minimal residual disease (MRD) using flow cytometry showed 0.6% myeloid precursor cells. PCR testing of *ETV6-ACSL6* in bone marrow found that the rearrangement ratio was 0.019%. Then, the patient received four cycles of cytarabine (2.5 g, by 3-h infusion every 12 h for 3 days), after which the *ETV6-ACSL6* rearrangement ratio was 0.246%. Since the rearrangement ratio increase was a sign of an uncontrolled disease, the patient received a haploidentical stem cell transplantation from her mother. Fifteen months after diagnosis, which was 6 months after stem cell infusion, the patient was still in a disease-free condition. MRD tests using both flow cytometry and PCR revealed no abnormal myeloid precursor cells and an undetectable rearrangement ratio. The patient’s WBC count was 6 × 10^9^/L, her hemoglobin level was 120 g/L, and her platelet count was 155 × 10^9^/L at the last follow-up.

### Review of the literature

Literature searches were performed in the Medline and Embase databases in July 2019 to retrieve articles related to the *ETV6-ACSL6* fusion gene. The search items included “ETV6-ACSL6” and “TEL-ACS2”. Ultimately, nine reports published between 1999 and 2016 involving 15 cases with detailed clinical data were identified and included for analysis. Detailed information for all the cases is summarized in Table 1.

**Table 1:** Characteristics of patients with *ETV6-ACSL6* rearrangement

No	Age	Sex	Diagnosis	Symptoms	WBC (× 10^**9**^/L)	Eos (%)	Baso (%)	Blasts (%)	Hb (g/L)	Plt (×10^**9**^/L)	BM	Cytogenetics	Rearrangement rate	Treatments	OS	Ref
1	68	F	RAEB	N/A	28.2	3	69	23	84	375	30.8% myeloblasts, 5.4% baso	t(5;12)(q31;p13)	N/A	Ara-C	0.75 month	[5]
2	27	M	AML	relapse of AML-M2	41.1	42	N/A	38	N/A	N/A	dry tap	t(5;12)(q31;p13)	N/A	chemotherapy (not specified)	1 month	[5]
3	53	M	AEL	back pain, fever, dyspnea	59.5	69	3	N/A	normal	141	3.2% myeloblasts, 11.2% promyelocytes, 49.2% matured eos	t(5;12)(q31;p13)	N/A	both steriod, cyclophosphamide, etoposide and Ara-C with VCR were ineffective;	11 months	[5]
														iDA: CR, relapsed in 2 months;		
														Inf-α		
4	49	M	aCML	N/A	N/A	Increased	N/A	N/A	N/A	N/A	N/A	t(5;12)(q31;p13)	N/A	N/A	N/A	[6]
5	29	M	PV	dyspnea	11.2	16	0	N/A	21	522	2% myeloblasts, 6% eos	t(5;12)(q23–31;p13)	13%	phlebotomy	> 42 months	[7]
6	31	F	PV to AML	N/A	18.2	N/A	N/A	N/A	13	278	PV 1% myeloblasts, 1% eos	t(5;12)(q23–31;p13)	4%	phlebotomy, splenic irradiation, purinethol for PV; Ara-C for AML	25 years after diagnosis of PV; 3 days after acutization	[7]
											AML 20% blasts, 2% baso					
7	44	M	MDS	N/A	22.7	39	42	N/A	55	85	N/A	t(5;12)(q31;p13), + 8	N/A	Ara-C, STI571, allo-SCT	> 8 months	[8]
8	42	M	MDS/MPN	N/A	N/A	N/A	N/A	N/A	N/A	N/A	N/A	t(5;12)(q31;p13)	N/A	N/A	N/A	[9]
9	55	M	secondary AML	N/A	N/A	N/A	N/A	N/A	N/A	N/A	N/A	t(5;12)(q31;p13)	90%	N/A	N/A	[2]
10	61	M	MPN	N/A	N/A	N/A	N/A	N/A	N/A	N/A	N/A	t(5;12)(q31;p13)	45%	N/A	N/A	[2]
11	51	M	CEL-NOS	dyspnea, palpitations	21.6	84	N/A	N/A	77	84	eos in various stages of maturation; no increase of blasts	t(5;12)(q31–33;p13)	80%	Imatinib, Inf-α	9 months	[3]
12	52	M	CEL-NOS	fatigue, muscle pain	N/A	13.3	N/A	N/A	mild anemia	normal	marked eos, no increase of blasts	t(5;12)(q31;p13)	45%	Imatinib	> 3 months	[10]
13–15	58 (38–71)	M (*n* = 2)	Eos-MPN (*n* = 2)	N/A	N/A	8.1 (2.3 to 62)	N/A	N/A	N/A	N/A	N/A	t(5;12) (*n* = 2)	N/A	Imatinib (*n* = 2), Sorafenib (*n* = 2)	64, 67 days (allo-SCT)	[11]
		F (*n* = 1)	secondary AML (*n* = 1)									complex karyotype with 5q and 12p (*n* = 1)		allo-SCT (*n* = 2)	7 months (without allo-SCT)	
16	37	F	CEL-NOS	fever, rashes, weight loss	42.4	53	28	N/A	70	24	7% myeloblasts, increased eos and baso	N/A	17.83%	DA, allo-SCT	15 months (still CR)	Present case

*RAEB* Refractory anemia with excess blasts, *AML* Acute myeloid leukemia, *AEL* Acute eosinophilic leukemia, *aCML* Atypical chronic myeloid leukemia, *PV* Polycythemia vera, *MDS* Myelodysplastic syndrome, *MPN* Myeloproliferative neoplasms, *CEL-NOS* Chronic eosinophilic leukemia, not otherwise specified, *WBC* White blood cell, *Eos* Eosinophils, *Baso* Basophils, *Hb* Hemoglobin, *Plt* Platelet, *BM* Bone marrow smear, *allo-SCT* Allogeneic hematopoietic stem cell transplantation, *Ara-C* Cytarabine, *iDA* Idarubicin, cytarabine, *Inf-α* Interferon-α, *DA* Daunorubicin, cytarabine, *OS* Overall survival, *CR* Complete response

## Results

The *ETV6-ACSL6* fusion gene was first reported by Yagasaki et al. in 1999 in three patients, with one presenting with refractory anemia with excess blasts (RAEB) with basophilia, one presenting with acute myeloid leukemia (AML), and one presenting with acute eosinophilic leukemia (AEL) [5]. Since then, the *ETV6-ACSL6* fusion gene has been confirmed by either fluorescence in situ hybridization (FISH), reverse transcription polymerase chain reaction (RT-PCR), or next generation sequencing (NGS). The present case is the 16th case reported so far. The demographic characteristics, clinical symptoms and diagnosis, treatment, and outcomes of the sixteen patients are listed in Table 1 [2, 3, 5–11]. Twelve patients were male, with a median age of 50 years (range, 27–71 years). Patients with *ETV6-ACSL6* fusion genes mainly presented with chronic eosinophilic leukemia, NOS (*n* = 5, 31%) and AML (*n* = 5, 31%). Among the patients with AML, one had acute erythroid leukemia, one had a second relapse of AML-M2, and three had secondary AML. The diagnoses of the remaining patients included two each with MDS, MDS/MPN, and MPN. Laboratory tests at diagnosis revealed that all patients had an increased WBC (*n* = 8), with a median count of 25.5 × 10^9^/L (range, 11.2–42.4 × 10^9^/L). Ten out of twelve patients (83%) had increased levels of eosinophils in peripheral blood, with a median level of 42% (range, 8.1–84%). Four out of five patients (80%) had increased levels of basophils (median, 28%; range, 3–69%); 6/8 had moderate to severe anemia (75%), while only 3/8 had reduced platelet counts. Cytogenetic tests revealed t(5;12)(q31;p13) in 9 of 15 patients, including one patient with + 8. The other two patients had t(5;12)(q23–31;p13), and one had t(5;12)(q31–33;p13). Of the remaining three patients reported by Gosenca et al. in 2009, two had t(5;12), and one presented with a complex karyotype involving 5q and 12p [11]. Treatments using tyrosine kinase inhibitors were ineffective, including imatinib (*n* = 4) and sorafenib (*n* = 2) [3, 10, 11]. Hydroxyurea was used in 58% of patients and sometimes led to temporary decreases in eosinophils and improvement of anemia and clinical symptoms [10]. Five patients received cytarabine with or without daunorubicin, and four patients received allogenic stem cell transplantation. The prognosis for patients with the *ETV6-ACSL6* fusion gene was rather poor, with seven patients dying within 1 year. Only one patient with polycythemia vera survived more than 42 months [7].

## Discussion

**Fig. 2:**
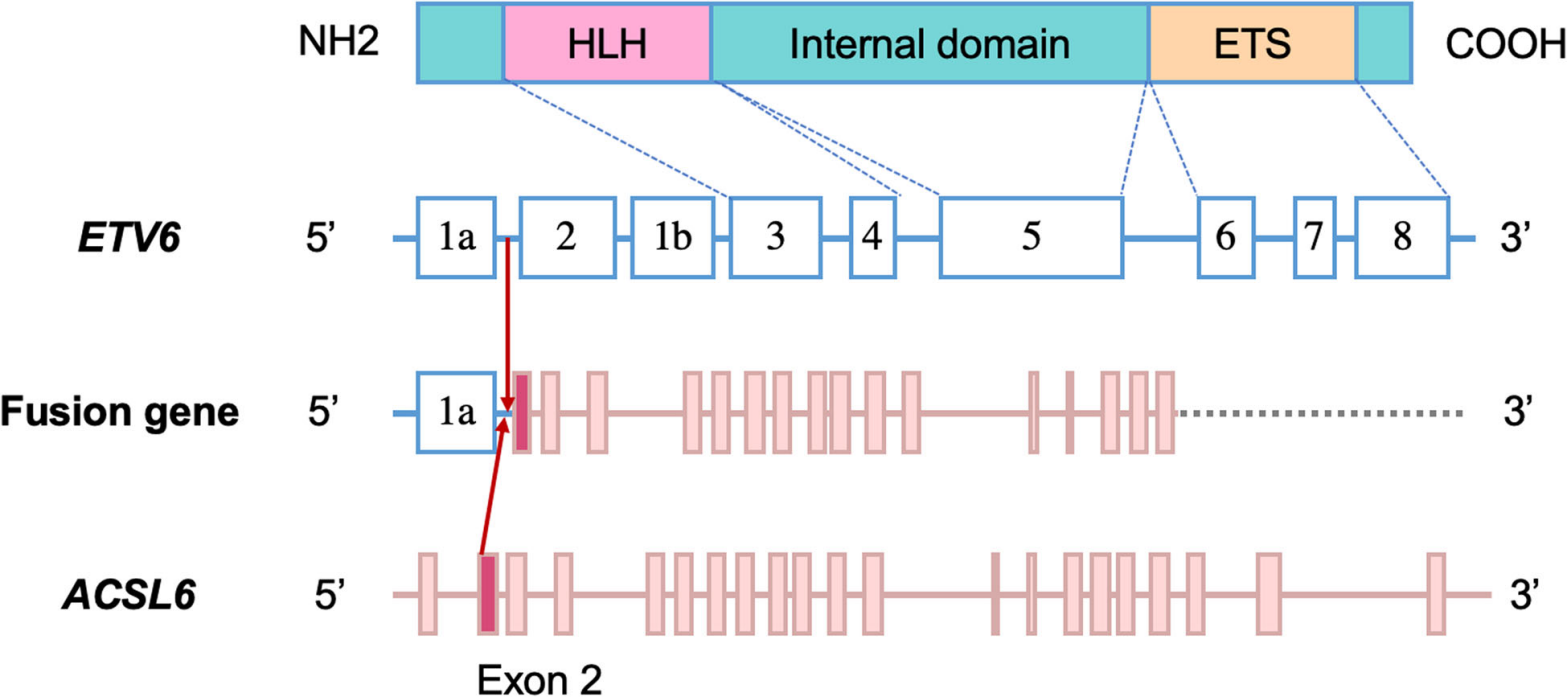
Schematic of the *ETV6* fusion with *ACSL6* in the present case. Red arrows represent the translocation site (between *ETV6* exon 1 and *ACSL6* exon 2). ETS, E-Twenty-Six domain; HLH, helix-loop-helix domain

ETV6 (ETS variant 6, previously known as TEL) is a transcription factor encoded by the ETS variant 6 gene (*ETV6*) belonging to the ETS (E-Twenty-Six) family, which is highly expressed in early hematopoietic progenitor cells [12, 13]. Deregulation of *ETV6* involves somatic and germline fusions, deletions, and rearrangements [14, 15]. *ACSL6* (Acyl-CoA synthetase long chain family member 6) encodes a long-chain acyl-CoA synthetase that catalyzes the formation of acyl-CoA from fatty acids, ATP, and CoA [16]. Different breakpoints lead to various in-frame and out-of-frame fusions of *ETV6* with *ACSL*6, which may cause leukemogenesis [5, 10]. A schematic of the *ETV6*-*ACSL6* fusion in the present case is shown in Fig. 2. The mechanisms by which *ETV6-ACSL6* gene fusion promotes leukemogenesis remain unclear. In general, *ETV6-ACSL6* fusion gene is very rare but can occur in myeloid neoplasms. Sixteen cases have been sporadically reported thus far. The incidence of the *ETV6-ACSL6* fusion gene might be underestimated. Several studies reported the recurrent translocation of t(5;12)(q31;p13); however, they did not perform molecular analysis to further verify whether it contained *ETV6-ACSL6* fusion [8, 17, 18].

In terms of the clinical characteristics, we found that the *ETV6-ACSL6* fusion gene seemed to occur predominantly in males. The median age was 50 years, which was slightly younger than that of patients with chronic myeloid disorders [19]. Of note, all patients with the fusion gene presented with myeloid disorders, with a slightly predominance of chronic myeloid disorders. Among patients with chronic myeloid disorders, many had chronic eosinophilic leukemia, NOS, and others suffered from MDS, MDS/MPN, and MPN. In addition, we found that the majority of the patients had increased eosinophils in peripheral blood [3, 5, 7]. Additionally, our study revealed that 80% of patients also had increased basophils in peripheral blood. Therefore, an increase in eosinophils and/or basophils seems to be a representative sign of the *ETV6-ACSL6* fusion gene. Nearly one-third of the patients were diagnosed with AML, of which more than half had secondary AML. In the present case, although the patient was diagnosed with chronic eosinophilic leukemia, NOS, she had a rapid progressive disease pattern with 7% myeloblasts in her bone marrow, accompanied by fever, skin rashes, and an enlarged liver and spleen. The patient would probably be unresponsive to conventional chemotherapy for CEL and may suffer from acute transformation into AML, which has a poor prognosis [20].

Overall, the prognosis of patients with the *ETV6-ACSL6* fusion gene is poor. Treatment strategies are varied due to the diverse clinical presentations as well as a lack of consensus guidelines for treatment. For the cases that presented as chronic eosinophilic leukemia, NOS, several reports indicated treatment with tyrosine kinase inhibitors was attempted, and all cases turned out to be nonresponsive. Unlike *ETV6-PDGFRB* or other rearrangements of *PDGFRB*, which are receptor-tyrosine kinase fusion genes, the *ETV6-ACSL6* fusion gene does not result in a fusion protein with constitutive tyrosine kinase activity [21]. Therefore, tyrosine kinase inhibitors are not recommended for these patients [22]. For patients presenting with acute leukemia, standardized intensive chemotherapy followed by allogenic stem cell transplantation might be successful. For the four patients who received allogenic stem cell transplantation, one died on day + 67 due to the relapse of secondary AML, and one died on day + 64 due to transplant complications [11]. The other two patients (Patient 7 and the present patient) survived more than 8 months and 15 months, respectively [8]. In the present case, although the patient was diagnosed with chronic eosinophilic leukemia, NOS, she experienced a rapidly progressive and severe clinical course. Hence, she received allogenic stem cell transplantation. At the 15-month follow-up, the patient was still in a disease-free condition.

## Conclusions

The *ETV6-ACSL6* fusion gene is extremely rare. Patients with the fusion gene mainly present with myeloproliferative and myelodysplastic disorders, typically chronic eosinophilic leukemia, NOS. The patients usually have increased eosinophils and/or basophils, and there is a very real possibility of transformation to AML. We recommend that intensive therapies should be an initial consideration for eligible patients. Meanwhile, the presence of MRD can be monitored in patients by flow cytometry as well as PCR for the *ETV6-ACSL6* rearrangement ratio. In the future, clinical trials and targeted therapies are needed to modify the natural history of the disease.

## Data Availability

All literature included in the manuscript is listed in the references. Articles were identified in the Medline and Embase databases, and access to the full texts was dependent on journal and institutional constraints.

## Abbreviations

NOS: Not otherwise specified
WBC: White blood cell
MRD: Minimal residual disease
RAEB: Refractory anemia with excess blasts
AML: Acute myeloid leukemia
AEL: Acute eosinophilic leukemia
FISH: Fluorescence in situ hybridization
RT-PCR: Reverse transcription polymerase chain reaction
NGS: Next-generation sequencing
